# Associations between hand osteoarthritis, obesity and lipid metabolism: a cross-sectional study of the Halland County Osteoarthritis (HALLOA) cohort

**DOI:** 10.1186/s12891-024-08073-x

**Published:** 2024-11-22

**Authors:** Elisabeth Brogren, Maria Andersson, Melker Westenius, Jenny Wittrup, Malin Zimmerman

**Affiliations:** 1 Department of Hand Surgery, Skåne University Hospital, Malmö, Sweden; 2 Department of Translational Medicine, Lund University, Lund, Sweden; 3 Department of Clinical Sciences, Rheumatology, Lund University, Lund, Sweden; 4 Spenshult Research and Development Centre, Halmstad, Sweden; 5 Bokskogen Primary Health Care Centre, Malmö, Sweden; 6 Department of Orthopedics, Helsingborg Hospital, Helsingborg, Sweden

**Keywords:** Osteoarthritis, Body mass index, Cholesterol, LDL, Cholesterol, HDL, Lipid metabolism, Obestity, Abdominal

## Abstract

**Background:**

To determine whether obesity and markers of lipid metabolism are associated with radiological hand osteoarthritis (OA) in the Halland County Osteoarthritis (HALLOA) cohort.

**Methods:**

In this cross-sectional study, we included 231 participants aged 30–65 from the HALLOA cohort, which began in 2017 and is ongoing. Hand OA was defined as ≥ 2 joint groups (distal interphalangeal, proximal interphalangeal, and carpometacarpal I) with Kellgren-Lawrence grade ≥ 2. The severity of hand OA was classified in terms of the number of affected joint groups (moderate hand OA 2–4 joint groups, severe hand OA 5–6 joint groups). Metabolic profile, including body mass index (BMI), bioimpedance, waist circumference, blood pressure, serum leptin, total cholesterol, low-density lipoprotein (LDL) cholesterol, high-density lipoprotein (HDL) cholesterol, and triglycerides, were obtained. Multicollinearity was assessed with Pearson’s correlation and associations with logistic regression analyses adjusting for age, HDL-cholesterol, and central obesity.

**Results:**

Two-thirds of the participants were women, and 91 (39%) had hand OA. We found a relationship between LDL-cholesterol and prevalent hand OA in women with an odds ratio of 1.7 (95% CI 1.1–2.6) and an association between LDL-cholesterol and severity of hand OA in women; odds ratio for no hand OA vs. moderate hand OA was 1.6 (95% CI 1.0-2.4) and for no hand OA vs. severe hand OA 2.5 (95% CI 1.2–4.9). There were no significant relationships between hand OA and obesity or serum leptin levels.

**Conclusion:**

Circulating LDL-cholesterol levels were associated with the prevalence and severity of hand OA in women but not men.

**Trial registration:**

ClinicalTrials. Gov (NCT04928170), Date of registration: 2017-12-20.

**Supplementary Information:**

The online version contains supplementary material available at 10.1186/s12891-024-08073-x.

## Background

Obesity is a significant risk factor for knee osteoarthritis (OA), attributed to mechanical stress beyond the physiological capabilities of the weight-bearing knee joint [1]. However, reported relationships between overweight and hand OA indicate that mechanical load related to body weight may not be the only factor responsible for the development of OA [2–5]. Associations between OA and several other metabolic diseases, such as diabetes type II, hypertension, cardiovascular disease, and lipid disturbances, support the concept of a metabolic syndrome-associated osteoarthritis (Met-OA), an obesity-linked phenotype where an increased metabolic stress seems to exacerbate knee and hand OA [4, 6–8].

Adipose tissue can be regarded as an endocrine organ, synthesizing adipokines known to activate low-grade inflammation [9]. Leptin has been suggested to play a critical role in the pathophysiology of Met-OA by mediating inflammatory and degradative effects on cartilage and synovia [10, 11]. Emerging in-vitro and animal model-based evidence also indicates that low-density lipoprotein (LDL) cholesterol could be involved in the pathophysiology of synovial inflammation, cartilage destruction, and bone deformation in OA [12, 13].

The increased metabolic stress involved in Met-OA is particularly suitable for study in hand joints, minimizing the confounding effect of excessive articular load. Previous systematic reviews and meta-analyses have found moderate evidence for a weakly positive association between weight or body mass index (BMI) and hand OA but with a substantial study heterogeneity [2, 3]. Central obesity predicts metabolic risk better than total body fat [14]. Despite this, BMI is commonly used in Met-OA studies, even though measures like waist circumference of visceral fat area (VFA) better capture central obesity [2, 3, 15].

In addition to obesity, age and female sex are substantial risk factors for hand OA [16]. It has been suggested that the aging OA phenotype usually presents above 65, whereas Met-OA typically affects the middle-aged [7]. Most studies on obesity and hand OA focus on elderly patients, potentially confounding the results [3, 16]. The risk of developing hand OA increases in women during menopause, although the pathophysiology and sex differences of OA prevalence remain poorly understood [16, 17].

This cross-sectional study aimed to assess the relationship between obesity, markers of lipid metabolism, and radiologically confirmed hand OA in a cohort of middle-aged men and women from the Halland osteoarthritis (HALLOA) study.

## Methods

### Study design and participants of HALLOA

The HALLOA study is a longitudinal cohort study in southwest Sweden that enrolled 306 middle-aged individuals with knee pain between 2017 and 2019. The inclusion criteria were: (1) current knee pain and (2) age between 30 and 65 years. The exclusion criteria were: (1) prior radiographic knee OA, (2) cruciate ligament rupture, and (3) rheumatological disorder. The participants in HALLOA were recruited when seeking care for knee pain in primary health care or by advertisements in local newspapers. The participants are examined at baseline, 2 and 5 years with clinical examination and followed yearly with blood samples. During the clinical examination, trained assessors examined the hands. Clinical hand OA was classified according to Altman with pain and stiffness in the hand as well as soft tissue swelling in ≥ 2 of 10 joints and < 3 swollen MCP-joints and soft tissue swelling in ≥ 2 DIP-joints or deformities in ≥ 2 of 10 joints as found at clinical examination [18]. Radiographs of both hands were obtained at 2 and 5 years. The HALLOA cohort is registered at ClinicalTrials.gov, NCT04928170, and has previously been described in detail [19].

### Participants of the current study

**Fig. 1:**
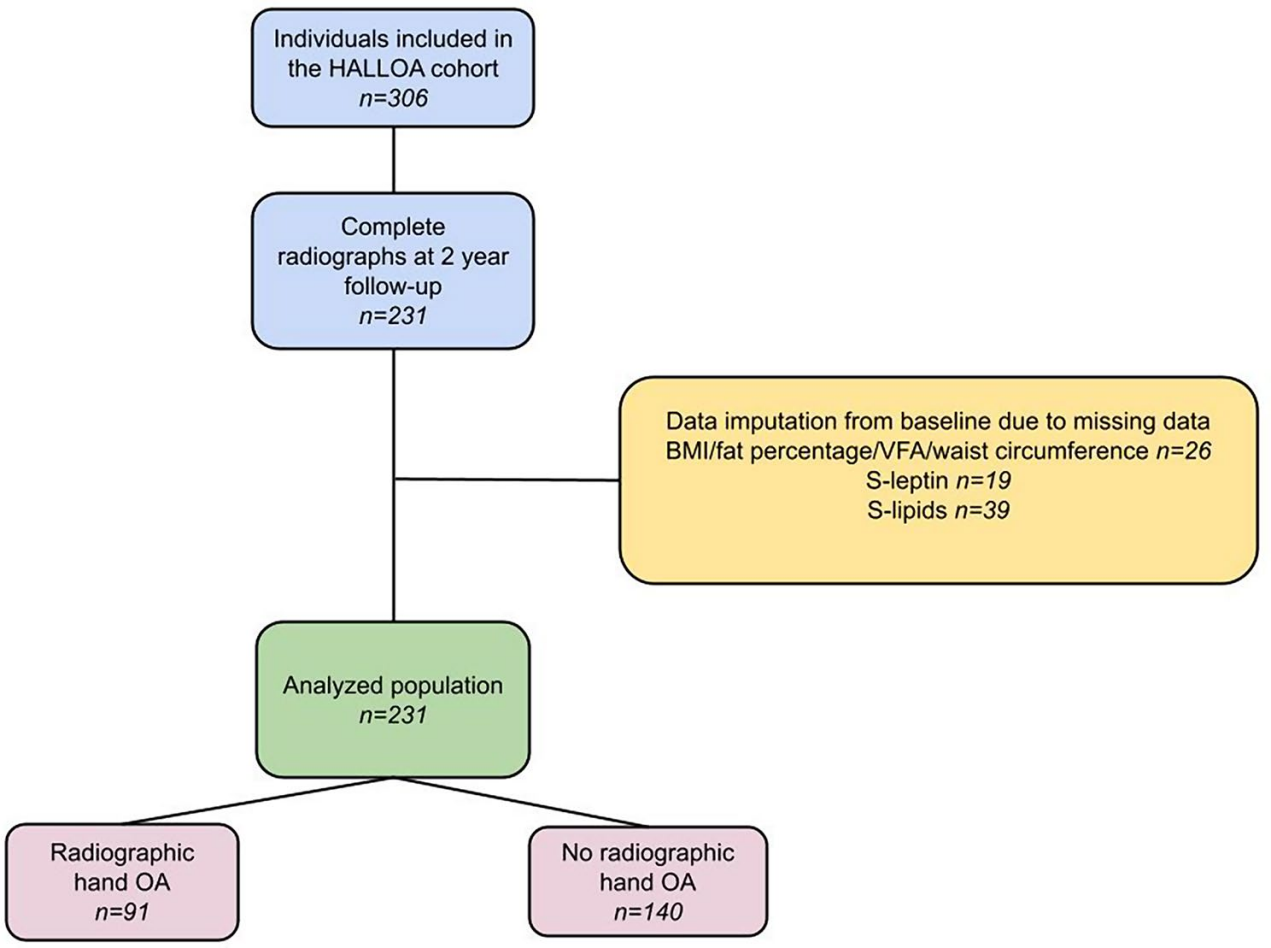
Flow chart describing the inclusion process

In this cross-sectional study of 2-year data obtained in the HALLOA study, we included participants who underwent radiographic examination of both hands at the 2-year follow-up (i.e., between Aug 20, 2020 and 31 Oct 2022). Participants were asked about their medical history and current use of statins and hypertensive- and diabetic medication at the 2-year follow-up. Since the majority of included participants were women, and since hand OA is more common in women, the results are presented stratified by sex. The inclusion process is illustrated in Fig. 1.

### Hand OA definition

Standard posteroanterior and oblique radiographs of both hands were obtained. Two assessors (EB and MZ) independently evaluated the radiographs and graded OA in hand joints according to the Kellgren-Lawrence (KL) classification [20]. Hand OA was defined as previously described by Dahaghin et al. [6].; the joints were grouped into distal interphalangeal (DIP) joints, proximal interphalangeal (PIP) joints, and first carpometacarpal (CMC) joints. A group was considered positive if at least one joint in the group was graded ≥ 2, according to KL. Hand OA was defined as having ≥ 2 in KL score in at least two of the three groups (DIP, PIP, and first CMC) in at least one hand [6]. Erosive OA was not specifically analyzed. The inter-rater agreement of classifying the participants as “no hand OA” versus “hand OA” was evaluated with Kappa statistics, showing a Kappa coefficient of 0.62 (95% CI 0.51–0.72), *p* < 0.001, indicating moderate agreement [21]. In cases of controversy, the radiographs were evaluated together by the two assessors to obtain consensus and uniformity.

To further assess for a dose-response relationship, hand OA was categorized as no hand OA if there was no OA or only one joint group was involved, moderate hand OA if 2–4 joint groups were involved, and severe hand OA if 5–6 joint groups were involved (i.e., DIP joints, PIP joints, and first CMC joint in both hands).

### Blood samples

Fasting venous blood samples were drawn at the clinical examination visit. Fasting plasma glucose (mmol/L), triglycerides (TG) (mmol/L), total cholesterol (mmol/L), high-density lipoprotein (HDL)-cholesterol and low-density lipoprotein (LDL)-cholesterol (mmol/L), haemoglobin A1c (HbA1c) (mmol/mol) and C-reactive protein (CRP) > 1.0 mg/L were measured from venous blood, by the current laboratory standards at Halland County Hospital in Halmstad, Sweden, accredited according to SS-EN ISO 15,189.

Glucose, TG, total cholesterol, HDL- and LDL-cholesterol were analysed with photometry (Cobas 8000, Roche). HbA1c was analysed with liquid chromatography (TOSOH G8), and CRP was analysed with turbidimetry (Cobas 8000, Roche). CRP below 1.0 mg/L was further explored by applying a sensitive CRP ELISA method (KA0238, Abnova, Taiwan) showing good agreement with CRP turbidimetry (data not shown). Serum-leptin was analyzed with an ELISA method (11-LEPHU-E01, Alpco, Salem, US).

### Clinical metabolic measures

Waist circumference was manually assessed with a measuring tape (cm) around the waist at the height of the navel by trained assessors. Central obesity was classified by the International Diabetes Federation (IDF) as a waist circumference of ≥ 94 cm in men and ≥ 80 cm in women [22]. Body length and weight were measured, and body mass index (BMI) was calculated. Overweight was defined as BMI ≥ 25 and obesity as BMI ≥ 30 [23]. The proportion of fat (%) and visceral fat area (VFA) were assessed by Inbody 770^®^, Seoul, Korea [24]. Raised VFA level was classified as having VFA ≥ 100cm^2^ [25]. Blood pressure was measured after five minutes of rest (Omron M3, Sundmed AB, Sweden). According to the World Health Organization, hypertension was classified as systolic blood pressure ≥ 140 mmHg and/or diastolic blood pressure ≥ 90 mmHg and/or treatment of previously diagnosed hypertension [26].

### Power analysis

Sample size calculations for the HALLOA cohort were based on four research areas, mainly focusing on metabolic risk factors in knee OA [19]. To find a difference in waist circumference of a mean of six (standard deviation 12) cm, with an effect size of 0.5 and power of 80%, 195 individuals were needed [19]. A new sample size calculation was performed for the current study based on previously reported odds ratios for obesity and hyperlipidemia on the development of hand OA [2, 27]. To find an expected odds ratio of 2, with an effect size of 0.5 and power of 80%, 112 individuals were needed.

### Statistical analyses

For continuous data, means, standard deviations (SD), medians, and interquartile ranges (IQR) were calculated after testing normal distribution with the Shapiro-Wilks test. The independent t-test or Mann-Whitney U test were used to compare continuous variables, and the Chi 2 test for proportions.

A total of 62 participants had missing data in terms of clinical measurements and/or blood samples at the 2-year follow-up, ranging from missing one value (one participant) to all values (five participants) (Table 1; Fig. 1). We compared the observed baseline data to the observed 2-year data for complete cases, showing similar values (data not shown). Thus, we assumed that the missing values were similar to the observed baseline values, and to yield a more complete data set, single imputation was performed for the 62 participants with missing data, where a participant’s baseline value (i.e., clinical measures or serum-lipids/serum-leptin levels at baseline) was carried forward and replaced the missing value at the 2-year follow-up [28].

**Table 1:** Characteristics of included participants stratified by sex and prevalence of radiographically confirmed hand osteoarthritis

	Women		Men	
	Hand OA *n* = 66	No Hand OA *n* = 89	Hand OA *n* = 25	No Hand OA *n* = 51
Age (years), md [IQR]	60 [57–61]	54 [47–59]	58 [55–63]	53 [45–59]
Clinical hand OA, n (%)	40 (60)	17 (19)	10 (40)	13 (25)
BMI (kg/m²), md [IQR]	26 [23–30]	25 [23–30]	27 [24–30]	27 [24–29]
BMI ≥ 25, n (%)	34 (52)	42 (47)	18 (72)	32 (63)
BMI ≥ 30, n (%)	13 (20)	22 (25)	6 (24)	9 (18)
Fat percentage, mean ± SD	34 ± 7	34 ± 8	24 ± 7	24 ± 7
VFA (cm²), md [IQR]	114 [77–170]	109 [73–161]	93 [71–130]	92 [65–123]
VFA ≥ 100 cm², n (%)	41 (63)	50 (55)	10 (40)	20 (40)
Waist circumference (cm), mean ± SD	94 ± 12	94 ± 16	101 ± 8	100 ± 10
Central obesity, n (%)	60 (91)	74 (83)	21 (84)	37 (73)
Serum-leptin (ng/ml), md [IQR]	18 [8.3–36]	21 [9.1–36]	5.3 [3.7–5.3]	7.3 [3.2–12]
Tot-c*, mean ± SD	5.7 ± 1.1	5.1 ± 1.0	5.2 ± 1.1	5.3 ± 1.2
Triglycerides*, md [IQR]	0.9 [0.7–1.3]	0.9 [0.7–1.1]	1.1 [0.8–1.5]	1.0 [0.7–1.4]
HDL-c*, mean ± SD	1.9 ± 0.42	1.8 ± 0.46	1.5 ± 0.37	1.4 ± 0.36
LDL-c*, mean ± SD	3.6 ± 1.0	3.1 ± 0.8	3.4 ± 1.0	3.6 ± 1.2
Statin use, n (%)	3 (5)	8 (10)	2 (9)	4 (9)
Diabetes, n (%)	1 (2)	4 (5)	0 (0)	1 (4)
Hypertension, n (%)	38 (58)	34 (38)	16 (32)	34 (68)

OA; Osteoarthritis, BMI; Body mass index, VFA; Visceral fat area, Tot-c; Total cholesterol, HDL-c; High-density lipoprotein cholesterol, LDL-c; Low-density lipoprotein cholesterol, md; Median, IQR; Interquartile range, SD; Standard deviation.*Given as mmol/l

Mean (SD) and median (IQR) are shown after single imputation of baseline data for 62 participants. Number of missing data was 26 participants for BMI, fat percentage, VFA and waist circumference, for serum-leptin 19, and for lipids 39 participants

To assess multicollinearity, pairwise correlations were performed between blood samples (total cholesterol, TG, HDL-cholesterol, LDL-cholesterol, and leptin) and measures for overweight (BMI, VFA, fat percentages, and waist circumference). If the Pearson correlation coefficient was < 0.7, the likelihood of collinearity was regarded as low, and the variable was further assessed in a univariate logistic regression analysis, with no hand OA/hand OA as the dependent variable. In cases of high likelihood of collinearity (Pearson correlation coefficient > 0.7), the variable that the authors judged as the most clinically relevant was chosen for further assessment in a univariate logistic regression analysis. Hence, since BMI correlated highly with VFA, increased VFA, waist circumference and fat percentage, BMI was chosen since it is the most commonly used measure of obesity in literature. Total cholesterol correlated highly with LDL-cholesterol, and since LDL-cholesterol was the most interesting variable based on previous research, LDL-cholesterol was chosen. The univariate analyses were performed for women and men separately, and if the p-value was < 0.3 in the univariate analysis, it was further analyzed in a multivariate logistic analysis adjusting for age (i.e.central obesity (dichotomous), HDL-cholesterol (continuous), LDL-cholesterol (continuous) and age (continuous) were included in a multivariate logistic analysis for women, and central obesity and age for men). As sensitivity analyses, we performed the same univariate logistic regression analyses for the complete case dataset as for the imputated dataset.

Finally, we performed a multinominal regression analysis adjusting for the same variables in women; age (continuous), central obesity (dichotomous), HDL-cholesterol (continuous), and LDL-cholesterol (continuous) to evaluate any dose-response relationship between these factors and the severity of hand OA. A p-value below 0.05 was used for statistical significance, and in all analyses, 95% CI was calculated when appropriate.

## Results

### Population characteristics

In total, 306 participants were enrolled in the original HALLOA cohort [19]. Of them, 231 participants (76 men and 155 women) underwent hand radiographs at the 2-year follow-up and were thus included in the current cross-sectional study. The participant characteristics are presented in Table 1. Ninety-one participants (39%) had radiologically confirmed hand OA, and 140 participants (61%) had no hand OA. Of the participants with radiological hand OA, 80 participants also had clinical hand OA (Table 1). The cohort was slightly overweight, and participants with hand OA were older than those without hand OA. Severe cardiovascular events were rare; angina pectoris was reported by one participant with hand OA and one participant with no hand OA, previous myocardial infarction by three participants with hand OA and two with no hand OA, cerebrovascular accidents by two participants with hand OA and deep venous thrombosis by two participants with hand OA and one with no hand OA.

### Non-participant analysis

The 75 non-participants (i.e., participants with missing hand radiographs at the 2-year follow-up) were younger than the participants but had a similar metabolic profile and sex distribution (Supplementary Table S_1).

### Factors associated with radiographic hand OA

Table 2 shows the results of Pearson’s pairwise correlations, where ≥0.7 was considered to have a high likelihood of collinearity. After assessment of multicollinearity, univariate logistic analyses evaluating associations between prevalent hand OA and BMI, increased VFA, central obesity, triglycerides, HDL-cholesterol, LDL-cholesterol, and S-leptin were performed for women and men separately (Table 3). The same analyses were performed for the complete case dataset, showing similar results as the imputated dataset (Supplementary Table S_2).

**Table 2:**
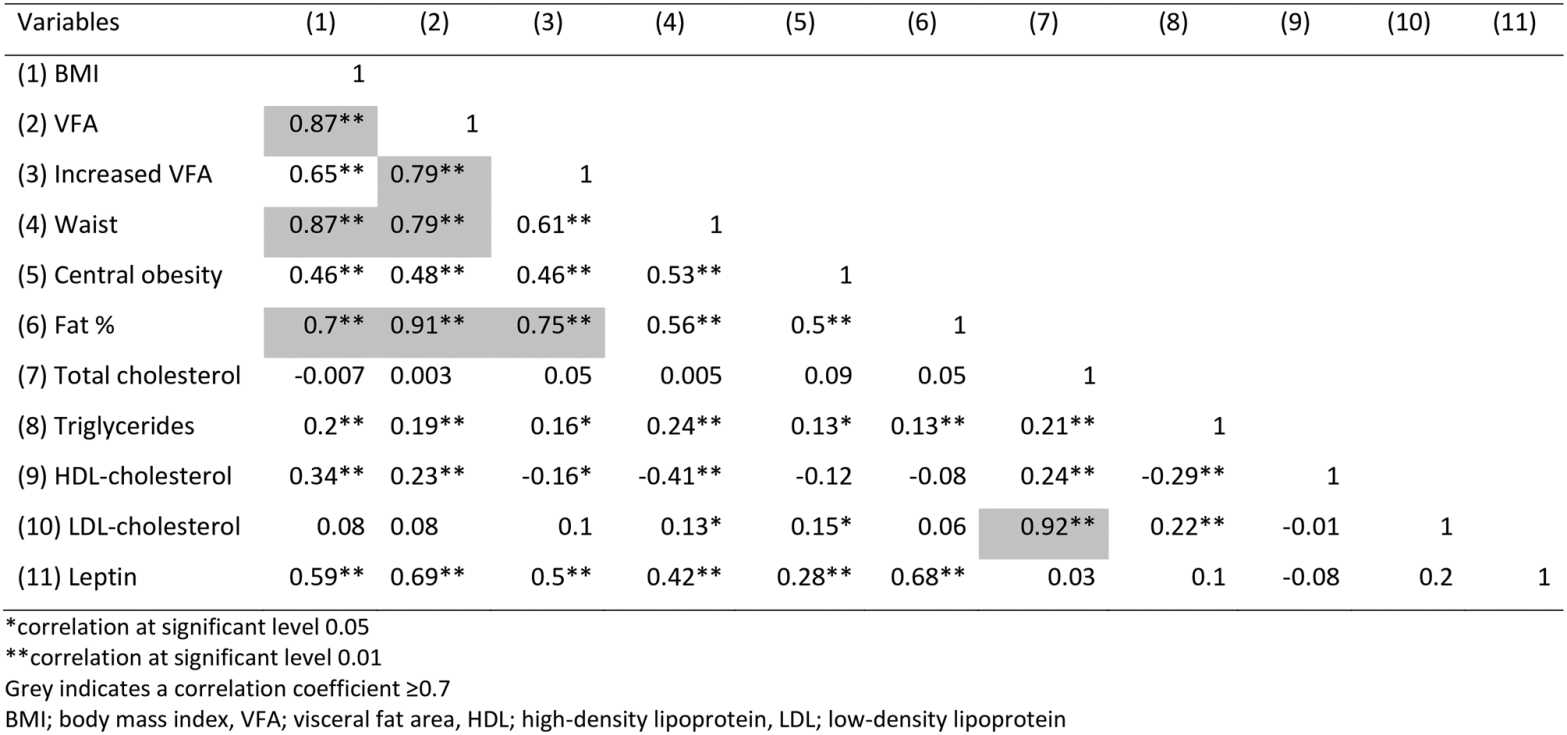
Correlation matrix of Pearson’s pairwise correlations to assess for collinearity of included metabolic factors

**Table 3:** Univariate logistic regression analyses on metabolic factors and radiological hand osteoarthritis, stratified by sex

	Women		Men	
	Odds ratio (95% CI)	*P*-value	Odds ratio (95% CI)	*P*-value
BMI (kg/m^2^)	1.00 (0.94–1.06)	0.95	1.05 (0.91–1.21)	0.50
No central obesity	*Reference*			
Central obesity	2.03 (0.74–5.54)	0.17	1.99 (0.58–6.82)	0.28
Normal VFA	*Reference*			
Increased VFA	1.33 (0.69–2.57)	0.39	1.00 (0.38–2.66)	1.00
TG (mmol/L)	1.02 (0.62–1.67)	0.94	1.02 (0.50–2.06)	0.96
HDL-c (mmol/L)	1.50 (0.73–3.09)	0.28	1.67 (0.44–6.32)	0.45
LDL-c (mmol/L)	1.93 (1.29–2.88)	**0.001**	0.99 (0.61–1.60)	0.96
Leptin (ng/ml)	1.00 (0.99–1.01)	0.67	0.98 (0.92–1.05)	0.64

BMI; Body mass index, VFA; Visceral fat area, TG; Triglycerides, HDL-c; High-density lipoprotein cholesterol, LDL-c; Low-density lipoprotein cholesterol

Central obesity was defined as a waist circumference of ≥ 94 cm in men and ≥ 80 cm in women. Increased VFA was defined as > 100 cm². P-values < 0.05 are marked as bold

In the next step, multivariate logistic regressions for women including central obesity, LDL-cholesterol, HDL-cholesterol, and age, and for men, including central obesity and age, were performed. Age was the only variable associated with hand OA in men, and age was also related to prevalent hand OA in women (Table 4). Among women, there was also a persistent association between hand OA and LDL-cholesterol levels in the multivariate logistic regression, with an odds ratio (OR) of 1.73 (95% CI 1.13–2.63; Table 4).

**Table 4:** Multivariate logistic regression analyses on metabolic factors and radiographic hand osteoarthritis, stratified by sex

	Women		Men	
	Odds ratio (95% CI)	*P*-value	Odds ratio (95% CI)	*P*-value
Age (years)	1.22 (1.12–1.32)	**< 0.001**	1.16 (1.06–1.27)	**0.001**
No central obesity	Reference			
Central obesity	3.00 (0.90-10.02)	0.075	1.20 (0.30–4.83)	0.79
HDL-c (mmol/L)	2.10 (0.86–5.14)	0.11	NA	
LDL-c (mmol/L)	1.73 (1.13–2.63)	**0.011**	NA	

HDL-c; High-density lipoprotein cholesterol, LDL-c; Low-density lipoprotein cholesterol. Central obesity was defined as a waist circumference of ≥ 94 cm in men and ≥ 80 cm in women. P-values < 0.05 are marked as bold. NA; Not applicable

### Associations between LDL-cholesterol and severity of hand OA in women

Unadjusted LDL-cholesterol levels in women with no hand OA was 3.1 (SD 0.8), in women with moderate hand OA 3.5 (SD 0.9), and in women with severe hand OA 3.9 (SD 1.0). Unadjusted HDL-cholesterol and triglyceride levels did not differ between groups with different severity of hand OA (Supplementary Table S_3). The multinominal regression analysis adjusting for age, central obesity and HDL-cholesterol showed that increased severity of hand OA in terms of the number of involved joints was significantly associated with LDL-cholesterol levels in women (no hand OA vs. moderate OA; OR 1.57 (95% CI 1.01–2.44) and no hand OA vs. severe OA; 2.47 (1.24–4.92), Table 5). This may indicate a dose-response relationship, since increasing LDL-cholesterol levels were associated with having moderate hand OA compared to no hand OA, and even stronger associated with having severe hand OA compared to no hand OA.

**Table 5:** Multivariate regression analysis of metabolic factors and severity of hand osteoarthritis in women

	No hand OA* vs. moderate hand OA**			No hand OA* vs. severe hand OA***		
	OR	95% CI	*p*-value	OR	95% CI	*p*-value
Age (years)	1.19	1.10–1.29	**< 0.001**	1.42	1.19–1.69	**< 0.001**
Central obesity	3.51	0.92–13.4	0.07	1.50	0.23–9.75	0.67
HDL-cholesterol	2.35	0.93–5.94	0.07	1.55	0.33–7.32	0.58
LDL-cholesterol	1.57	1.01–2.44	**0.04**	2.47	1.24–4.92	**0.01**

OA; Osteoarthritis, OR; Odds ratio, CI; Confidence interval, HDL; High-density lipoprotein, LDL; Low-density lipoprotein

*No hand OA; 0–1 joint groups with radiological OA (*n* = 88)

**Moderate OA; 2–4 joint groups with radiological OA (*n* = 50)

***Severe OA; 5–6 joint groups with radiological OA (*n* = 15)

## Discussion

In this cross-sectional cohort study, higher levels of LDL-cholesterol were associated with prevalent radiological hand OA in women but not men. Moreover, we found a relationship between increased severity of hand OA, defined as the number of affected joints, and higher levels of circulating LDL-cholesterol among women. The odds for female participants in our cohort of having hand OA increased by 70% per mmol/L LDL-cholesterol, indicating that LDL-cholesterol may play a role in the pathophysiology of hand OA in middle-aged women and is an interesting target for further research.

The pathophysiological mechanisms involved in the development of Met-OA have gained increased research attention over the last decade, where both experimental and epidemiological studies have suggested a link between lipid metabolism and OA [12, 27, 29]. Gierman et al. showed that dietary cholesterol intake induced cartilage damage and knee OA development in mice independently of body weight and that statins may have a protective effect [30]. In a recent systematic review and meta-analysis, the authors found a twofold increase of dyslipidemia among patients with knee and hand OA compared to patients without OA [27]. Our findings are also consistent with the results from a more recent registry-based study of patients aged 30–89 years by Frey et al., where dyslipidemia was associated with higher odds ratios of hand OA in both men and women, with slightly higher odds ratios in women, irrespective of BMI [31]. Interestingly, the strongest association was found among the younger patients (29–49 years), with an OR of 1.72 [31].

High levels of cholesterol, especially oxidized LDL-cholesterol, and the involvement of macrophages, endothelial cells, and fibroblasts are well-studied features of atherosclerosis that have been suggested to play a role also in OA pathology [12, 32]. Even if dyslipidemia seems associated with knee and hand OA, previous evidence of a relationship between OA pathology and LDL-cholesterol in particular, is mainly based on preclinical studies [12]. In two smaller comparative analyses, individuals with knee OA had higher LDL-cholesterol serum levels than matched healthy controls [33, 34]. Our findings of an association between serum LDL-cholesterol and prevalence of hand OA among middle-aged women are, to our knowledge, the first to show this relationship in non-weight-bearing joints and a larger cohort. Further supporting the association is the fact that hand OA is associated with cardiovascular disease, which indicates a common low-grade inflammatory state, perhaps involving the same cell types [12, 35–39]. Coexistence between erosive hand OA and subclinical atherosclerosis was reported by Koutroumpas et al. in a small case-control study [36]. In the Framingham heart study of 1348 participants, symptomatic hand OA, but not radiographic hand OA, was associated with an increased risk of cardiovascular events [37]. Cemeroglu et al. reported that radiographically confirmed hand OA was associated with coronary artery stenosis in older women [38], and in a large study from Reykjavik, the authors found a linear relationship between the severity of hand OA and atherosclerosis in older women [39]. In addition to a relationship between LDL-cholesterol and hand OA, the female participants with hand OA in our cohort more often had hypertension than their counterparts without hand OA, which aligns with these previous reports.

A clinically important feature of Met-OA is the association with modifiable risk factors. The results from our study, as well as others [35, 39], implicate that hand OA in women may be a sign of underlying lipid disturbances that should be screened for and managed to prevent future cardiovascular events. Whether statin treatment can also reduce the risk of hand OA incidence and progression is disputed, and the evidence is limited [40]. Although a few studies have shown that statins may be protective against the progression of knee OA in patients with simultaneous Herbeden nodules [41, 42], a recent systematic review and meta-analysis of more than 6,000,000 patients reported that statin use was associated with increased OA development [43]. However, this review was flawed by including multiple retrospective studies, unclear definitions of statin use, and no information on treatment compliance [43]. Only a few participants in our study were prescribed statins; thus, meaningful analyses were impossible.

We found a modest to strong relationship between hand OA and central obesity in women, although it was not statistically significant. The wide 95% confidence interval indicates that our study could have been undersized and that a larger study would have shown more clear results.

Previous meta-analyses have suggested a weak positive relationship between hand OA and overweight. The inconsistency in included studies indicates that it may not be the obesity itself but rather the metabolic risk factors related to the lipid metabolism that play a role in hand OA development [2, 3]. In fact, a significant proportion of individuals classified as obese do not exhibit metabolic disorders [44, 45]. This subgroup has been termed metabolically healthy obese, in contrast to metabolically unhealthy obese, and is characterized by the absence of cardiovascular disease, insulin resistance, dyslipidemia, and hypertension [45]. In addition, metabolically unhealthy normal-weight individuals have increased levels of circulating lipids and increased risk of cardiovascular events despite normal weight, indicating that it is not obesity per se that increases the risk of dyslipidemia, but it is instead the dyslipidemia that is driving the metabolic diseases [44]. This phenomenon was reflected in our material, where lipid levels correlated weakly or not at all with obesity-related measures.

Metabolically healthy obesity is considered a transient state that tends to deteriorate towards metabolically unhealthy obesity over time [45]. Aging women are especially vulnerable to changes in metabolic status and body composition, with increasing central obesity, LDL-cholesterol levels, and cardiovascular risk during the menopausal transition [46, 47]. There are several proposed mechanisms by which the menopausal transition contributes to higher LDL-cholesterol levels. The estrogens are synthezied from cholesterol, of which the most potent form, estradiol (E2), is primarily produced in the ovaries of premenopausal women [48]. A decreased cholesterol consumption in the ovaries has been suggested as a possible explanation for the changes in lipid metabolism after menopause [46]. Other hypotheses include decreased postmenopausal LDL-receptor activity leading to higher levels of circulating LDL-cholesterol [49] and reported variations in the apolipoprotein genotype that can influence postmenopausal circulating cholesterol levels [50]. Estrogens may also have anti-oxidant properties, perhaps inhibiting the oxidation of LDL-cholesterol to ox-LDL, the form believed to play an essential role in the pathogenesis of atherosclerosis [49]. Postmenopausal estrogen replacement has a well-documented cardioprotective effect mainly by maintaining a more favorable lipid profile, especially if introduced within the first years of menopause and if the treatment is continued for a prolonged time [51]. Whether hormonal therapy can also affect the incidence, prevalence, or progression of hand OA in women by influencing lipid metabolism is currently unknown. However, the need for well-designed, placebo-controlled randomized studies in order to draw any conclusions regarding the protective role of hormonal therapy has been acknowledged and justified [52, 53]. Menopausal status and hormonal treatment were not recorded at the 2-year follow-up in the HALLOA cohort; thus, these factors could not be analyzed.

We did not find evidence to support the hypothesis that hand OA is associated with serum leptin. Previous data on the relationship between leptin and OA have mainly originated from experimental models and studies on knee OA [54]. In the NHANES-III cross-sectional study of 2477 patients, there was no association between serum leptin levels and hand OA [55]. Similar results were reported by Yusuf et al., who found no association between serum leptin and the progression of hand OA in 164 patients [56]. It is possible that analyses of leptin in synovial fluid from hand joints would show a different result, but the role of leptin in hand OA is more controversial than in knee OA [54].

The major strengths of this study are the well-defined and thoroughly investigated cohort and two of the authors independently assessed all radiographs and graded the level of OA. This study also has several limitations. Due to the cross-sectional study design, we could not assess the incidence, progression, or causality of hand OA in relation to obesity and lipid metabolism markers. The results from this study should thus be interpreted with caution since the possible exposure (central obesity and LDL-cholesterol levels) and outcome (hand OA) were measured at the same time. Well-designed longitudinal cohort studies are needed to explore potential associations further. In addition, the risk of type II error should also be considered, especially regarding the interpretation of the relationship between hand OA and central obesity in women. Since the HALLOA cohort consists of individuals who actively applied to participate, there is also a risk of selection bias. Participating individuals may be healthier than the average population and care more about their health. This might have affected our results since the association between hand OA and metabolic factors may not be linear but rather dose-dependent, and some of our results may hence be false negatives in this healthy population. Another potential bias concerns missing data. Variables were missing for 62 participants. Since weight, fat distribution, lipid- or leptin levels was unlikely to have changed substantially over the 2-year period among the participants with missing values, we assumed that the observed value at baseline could replace the 2-year data (single imputation). This might have resulted in underestimating the true variability or induced bias because missingness may be related to the unobserved values. We, however, also performed the univariate logistic regression analyses with the complete case data set and found similar outcome (Supplementary Table 2). By using a p-value-based strategy for including variables in the multivariate analyses, there is a risk of leaving out one or more relevant variables. Even if arbitrary, a p-value threshold of < 0.3 limits the risk of omitted-variable bias. This was also illustrated by the confidence intervals of variables not included in the multivariate logistic regression analyses (Table 3). The inclusion criteria excluded individuals above 65 years, which may have affected the results since hand OA prevalence increases with age. However, age was adjusted for in the multivariate regression analyses and did not substantially affect our findings of a relationship between LDL-cholesterol and hand OA in women. Finally, the definition of hand OA in this study was based solely on radiological features of OA in the CMC-, DIP-, and PIP joints. It did not include information on clinical examination, morning stiffness, or symptom-structure concordance as suggested by the newly launched 2023 EULAR criteria [57]. We might have underdiagnosed hand OA in our cohort using only radiological findings, although individuals meeting the 2023 EULAR criteria are likely to present with radiographic evidence in their CMC- and IP joints [57].

In summary, the results of this study needs validation in future longitudinal studies. However, the relationship between circulating LDL cholesterol and hand OA is an interesting direction for future research, with possible clinical implications such as screening of middle-aged women with hand OA for lipid disturbances and cardiovascular comorbidities.

## Conclusions

In our material, cross-sectional LDL-cholesterol levels were associated with prevalent hand OA in women but not men. In women, there was also a dose-dependent relationship between LDL-cholesterol levels and the severity of hand OA defined as the number of affected joints.

## Electronic supplementary material

Below is the link to the electronic supplementary material.

Supplementary Table S_1

Supplementary Table S_2

Supplementary Table S_3

## Data Availability

Data is provided within the manuscript and supplementary tables.

## Abbreviations

BMI: Body Mass Index
CI: Confidence Intervals
HALLOA: Halland County Osteoarthritis study
HDL: High-density lipoorotein
IQR: Interquartile Range
LDL: Low-density lipoprotein
Met-OA: Metabolic syndrome-associated osteoarthritis
NHANES-III: Third National Health and Nutrition Examination Survey
OA: Osteoarthritis
S: Serum
SD: Standard Deviation
VFA: Visceral Fat Area
