# Supplementary material for: Associations between hand osteoarthritis, obesity and lipid metabolism: a cross-sectional study of the Halland County Osteoarthritis (HALLOA) cohort
Source: BMC Musculoskelet Disord. 2024 Nov 22;25:944. doi: 10.1186/s12891-024-08073-x (PMC11583440; doi:10.1186/s12891-024-08073-x)
Supplement: Supplementary file 1 — Supplementary Table S_1 [file 12891_2024_8073_MOESM1_ESM.docx]

Supplementary Table S_1. Characteristics at inclusion of participants and non-participants (i.e., did not undergo hand radiographs at the 2-year follow-up)

|  | Participants  n=231 | Non-participants  n=75 |
| --- | --- | --- |
| Women, n (%) | 155 (67) | 57 (76) |
| Age at inclusion (years) | 54 [48-58] | 52 [42-57] |
| BMI (kg/m²) | 26 [23-29] | 26 [23-29] |
| Waist circumference (cm) | 95±13 | 95±14 |
| Central obesity, n (%) | 181 (77) | 54 (78) |
| Total cholesterol | 5.5±1.1 | 5.5±1.2 |
| Triglycerides | 0.9 [0.7-1.2] | 0.9 [0.7-1.4] |
| HDL-cholesterol | 1.7±0.47 | 1.7±0.49 |
| LDL-cholesterol | 3.4±1.1 | 3.5±1.1 |
| Systolic BP (mmHg) | 132±17 | 130±19 |
| Diastolic BP (mmHg) | 87±12 | 85±11 |

BMI; body mass index, HDL; high-density lipoprotein, LDL; low-density lipoprotein BP; blood pressure
