# Supplementary material for: Associations between hand osteoarthritis, obesity and lipid metabolism: a cross-sectional study of the Halland County Osteoarthritis (HALLOA) cohort
Source: BMC Musculoskelet Disord. 2024 Nov 22;25:944. doi: 10.1186/s12891-024-08073-x (PMC11583440; doi:10.1186/s12891-024-08073-x)
Supplement: Supplementary file 2 — Supplementary Table S_2 [file 12891_2024_8073_MOESM2_ESM.docx]

Supplementary Table S_2. Univariate logistic regression analyses on metabolic factors and radiological hand osteoarthritis, stratified by sex in the complete case dataset

|  | Women | | Men | |
| --- | --- | --- | --- | --- |
|  | Odds ratio (95% CI) | P-value | Odds ratio (95% CI) | P-value |
| BMI (kg/m^2^) | 0.99 (0.92-1.1) | 0.71 | 1.01 (0.88-1.21) | 0.76 |
| No central obesity | *Reference* |  |  |  |
| Central obesity | 2.52 (0.85-7.45) | 0.10 | 1.82 (0.52-6.41) | 0.35 |
| Normal VFA | *Reference* |  |  |  |
| Increased VFA | 1.40 (0.70-2.80) | 0.34 | 0.96 (0.35-2.7) | 0.34 |
| TRG (mmol/L) | 1.13 (0.65-2.0) | 0.66 | 0.99 (0.43-2.25) | 0.98 |
| HDL-c (mmol/L) | 1.42 (0.66-3.05) | 0.38 | 1.25 (0.27-5.7) | 0.77 |
| LDL-c (mmol/L) | 1.81 (1.25-2.85) | **0.002** | 0.77 (0.44-1.35) | 0.34 |
| Leptin (ng/ml) | 1.00 (0.99-1.01) | 0.67 | 0.98 (0.92-1.05) | 0.64 |

Number of missing data was 26 individuals for BMI, fat percentage, VFA and waist circumference, for serum-leptin 19, and for lipids 39 individuals.
