# Supplementary material for: Associations between hand osteoarthritis, obesity and lipid metabolism: a cross-sectional study of the Halland County Osteoarthritis (HALLOA) cohort
Source: BMC Musculoskelet Disord. 2024 Nov 22;25:944. doi: 10.1186/s12891-024-08073-x (PMC11583440; doi:10.1186/s12891-024-08073-x)
Supplement: Supplementary file 3 — Supplementary Table S_3 [file 12891_2024_8073_MOESM3_ESM.docx]

Supplementary Table S_3. Lipid profile in women stratified by severity of hand OA as number of involved joint groups

|  | No hand OA*  (n=88) | Moderate hand OA**  (n=50) | Severe hand OA***  (n=15) |
| --- | --- | --- | --- |
| Total cholesterol | 5.1±1.0 | 5.6±1.0 | 5.9±1.0 |
| Triglycerides | 0.9 [0.7-1.1] | 0.9 [0.7-1.2] | 1.2 [0.7-1.6] |
| HDL-cholesterol | 1.8±0.5 | 1.9±0.5 | 1.8±0.5 |
| LDL-cholesterol | 3.1±0.8 | 3.5±0.9 | 3.9±1.0 |

OA; osteoarthritis, HDL; high-density lipoprotein, LDL; low-density lipoprotein. All lipids were measured in mmol/L.

*No hand OA; involving 0-1 joint groups

**Moderate hand OA; involving 2-4 joint groups

***Severe hand OA; involving 5-6 joint groups
